# Proof-of-Concept for Adjusted Surface Energies and Modified Fines as a Novel Concept in Particle Engineering for DPI Formulations

**DOI:** 10.3390/pharmaceutics14050951

**Published:** 2022-04-28

**Authors:** Nicholas Bungert, Mirjam Kobler, Regina Scherließ

**Affiliations:** 1 Department of Pharmaceutics and Biopharmaceutics, Kiel University, 24118 Kiel, Germany; nbungert@pharmazie.uni-kiel.de; 2 Meggle Excipients and Technology, 83512 Wasserburg, Germany; mirjam.kobler@meggle.com

**Keywords:** carrier-based blend, inverse gas chromatography, ternary blends, co-milling, dry particle coating, respiratory, formulation development

## Abstract

Currently marketed dry powder inhaler (DPI) medicine lacks drug delivery performance due to insufficient powder dispersion. In carrier-based blends, incomplete drug detachment is typically attributed to excessive adhesion forces between carrier and drug particles. Adding force control agents (FCA) is known to increase drug detachment. Several researchers accounted this effect to a decrease in carrier surface energy (SE). In turn, an increase in SE should impede drug detachment. In this proof-of-concept study, we investigated the influence of the SE of the carrier material in binary blends by intentionally inverting the FCA approach. We increased SEs by dry particle coating utilising high-shear mixing, which resulted in decreased respirable fractions of the respective blends. Thus, we confirmed the SE of the carrier influences drug delivery and should be considered in formulation approaches. Complementing engineering techniques on the carrier level, we evaluated a method to modify the SE of extrinsic fines in ternary powder blends for inhalation. By the co-milling of fine lactose and an additive, we tailored the SE and hence the adhesiveness of additional fine excipients. Thus, the extent and the strength of drug–fines agglomerates may be controllable. For ternary DPI formulations, this work highlights the potential benefits of matching the SE of both fines and drugs.

## 1. Introduction

Dry powder drug delivery into the deep lungs is a challenging task. The branching of pulmonary structures with decreasing diameters needs drug particles to be micronised, typically smaller than 5 µm. Micronisation increases surface area and surface energy [1], which leads to high co- and adhesiveness. To enable reproducible dosing, scientists combine the drug with a coarser carrier, formulating interactive blends or adhesive mixtures [2]. The adhesion between drug and carrier is typically excessively strong, resulting in incomplete drug detachment from the carrier. Force control agents (FCA) are a well-known tool in carrier-based dry powder inhaler (DPI) formulation development [3,4,5]. They shall decrease the adhesion between drug and carrier particles in adhesive mixtures for inhalation and hence increase drug detachment during inhalation. Magnesium stearate is one of the best-studied additives for DPI application [6,7,8,9]. It is FDA (Food and Drug Administration) approved as safe for pulmonary application and already utilised in marketed products (e.g., Breo^®^ Ellipta^®^) [10]. Due to the complexity of particle–particle interactions in adhesive mixtures, the underlying principle of FCAs remains partly unsolved. It is reported that the mechanism of action bases on a combination of carrier smoothening [11] and the decrease of adhesion forces [12]. However, it remains challenging to distinguish which principle primarily causes the specific effect. Fundamental adhesion properties (thermodynamic adhesion, surface energy interaction) are determinable by inverse gas chromatography. Several researchers found the surface energy of magnesium stearate-treated powders to be decreased compared to untreated material [13]. Since magnesium stearate is the only well-investigated example, it remains unclear if this is a causal relationship and valid for other additives.

The aim of this study was to highlight the surface energy of solids as a main influence in adhesion, which will translate into an influence on aerodynamic performance. Herewith, we investigated if the observed effects of modified DPI carrier surface energies (SE) on respirable fractions are invertible. Furthermore, we examined particle engineering towards the tailored SE of fine excipients and their respective influence.

As a proof-of-concept, we prepared DPI carrier particles with artificially increased SEs by dry particle coating. As a continuation of the published data [14], we used previously applied methods and compared the SE impact of carriers with high SEs with carriers being treated with magnesium stearate (decreased SEs). According to the hypothesis that aerodynamic performance of adhesive blends increases with decreasing carrier SE (e.g., after processing with magnesium stearate), we expect the contrary for the high-energy carriers. The second part of this study translates this finding to fine excipients in ternary blends for inhalation. Ternary powder blends are DPI formulations, which contain additional fine excipient fractions (fines) to improve drug detachment and hence drug delivery. Grasmeijer et al., as well as Jones and Price, extensively reviewed the principles with which additional fines are likely to improve aerodynamic performances [15,16]. One of the main theories is the formation of agglomerates of the drug and fines. These agglomerates are prone to detach more easily from the carrier compared to the pure drug [17]. Furthermore, fines may adhere to higher-energy binding sites on the carrier, forcing the drug to bind on weaker adhesion spots. In 2021, we published a study on the influence of SEs in ternary powder blends. We found high SE fines to be superior and concluded that a higher SE will preferentially lead to more agglomeration with the drug and consequently higher drug detachment from the carrier. The different fine qualities originated from different manufacturing routes or storage conditions [18]. To evaluate and substantiate this theory, we engineered particles in the size level of fine lactose by co-milling. The resulting novel compound fines in this study were then assessed for their performance-affecting properties. Apart from specific SE modifications, the effect of intrinsic SE levels of different drugs is also part of this work. The surface energy interaction depends always on both interacting surfaces, emphasising the importance of the drugs’ surface energy level.

We assume that there is an optimum of SE interaction in dependency of the drug. When the interaction strength exceeds an optimum, we postulate that the drug-fines agglomerates do not disperse sufficiently during inhalation.

## 2. Materials and Methods

### 2.1. Materials

The first section of this study covers the carrier modification of a specific respiratory grade, sieved lactose quality, namely InhaLac^®^ 230 (IH230, Meggle, Wasserburg, Germany), using magnesium stearate (MgSt, Parteck^®^ LUB MST, Merck, Darmstadt, Germany) and poloxamer 188 (Pol, Lutrol^®^ micro 68, BASF, Ludwigshafen, Germany). To produce engineered fine excipients, we used the micronised, respiratory-grade lactose InhaLac 400 (IH400, Meggle) in combination with MgSt or Pol. IH230, after the removal of the present intrinsic fines (IH230rF), served as carrier material for ternary blends. Ipratropium bromide (d_90_ < 5 µm, Boehringer Ingelheim, Ingelheim, Germany) and fenoterol hydrobromide (d_90_ < 5 µm, Boehringer Ingelheim) were used as model drugs.

### 2.2. Methods

#### 2.2.1. Particle Size Distributions

Laser diffraction measurements provided particle size distributions (PSD) of all used excipients. We used the Helium Laser Optical System (HELOS^®^, Sympatec, Clausthal-Zellerfeld, Germany) equipped with the dry dispersion unit (RODOS^®^) and an automatic feeder (VIBRI^®^). The samples were dispersed by pressurised air (primary dispersion pressure of 2 bar for carrier samples and 3 bar for extrinsic fines). Depending on the sample, we used either an R1, R3 or an R4 lens. All data are shown as average of triplicate measurements. The respective standard deviation is depicted accordingly.

#### 2.2.2. Particle Engineering of DPI Carriers

We modified the carrier lactose using dry particle coating in the high-shear mixing unit of the Picoline^®^ (Picomix^®^, Hosokawa Alpine, Augsburg, Germany). The process comprised multiple steps. First, pre-sieving of additive (mesh size: 180 µm) and carrier (mesh size: 250 µm). Second, weighing both substances into the mixing vessel using the sandwich method. Third, a coating period of 15 min at 500 rpm. Each batch was 30 g containing 2% (*w*/*w*) of the additive, in line with earlier studies [14]. The dry particle coating was conducted at monitored lab conditions (21 °C ± 5 °C; 45% RH ± 15%).

#### 2.2.3. Removal of Intrinsic Fines

To exclude the effect of present intrinsic fines in ternary blends, we removed particles <32 µm (equates the mesh size) from IH230 using an air jet sieving technique (e200LS^®^, Hosokawa Alpine) at 4 kPa negative pressure for 29 min. The resulting material (IH230rF) served as carrier material for ternary interactive blends containing engineered fines.

#### 2.2.4. Particle Engineering of Extrinsic Fines

Particle engineering of fine lactose was conducted by co-milling using the Jet-O-Mizer^®^ (Fluid Energy Processing and Equipment Company, Bethlehem Pike Telford, PA, USA) air jet mill. We premixed IH400 with the respective additive (MgSt or Pol) at 10% (*w*/*w*) concentration for 45 min at 42 rpm in the Turbula^®^ blender (Willy A. Bachofen Maschinenfabrik, Basel, Switzerland) prior to co-milling.

The obtained pre-blends were fed manually into the air jet mill. The feed pressure was 9 bar for all milling procedures. The grinding pressure and the number of grinding cycles was adjusted to fit the PSD of IH400 being processed at 9 bar grinding pressure.

In order to meet these PSD requirements (InhaLac 400 after one grinding cycle at 9 bar), IH400 + 10% MgSt needed one grinding cycle at 7.5 bar, whereas IH400 + 10% Pol needed two cycles: the first at 9 bar and a second at 7 bar grinding pressure. The batch size of the co-milling was approximately 10 g, and the milling was performed at monitored lab conditions (21 °C ± 5 °C; 45% RH ± 15%).

#### 2.2.5. Surface Area and Surface Energy Determination

To determine the specific surface area (SSA) and SE distributions of all materials, we used the Surface Energy Analyser (SEA, Surface measurement Systems, London, UK), an inverse gas chromatograph (iGC). The sample preparation included transferring the powder into silanised glass columns (4 mm inner diameter for carriers and 3 mm inner diameter for drugs and fines) and fixing the sample on both ends using silanised glass wool. The magnesium stearate processed fines needed a stepwise column filling. Due to their greasy nature, we inserted multiple glass wool spacers between small amounts of sample (approx. one spacer every 25 mg). A tapping procedure using the SMS Column Packer Accessory (Surface Measurement Systems) excluded voids within the sample columns. Experiments were performed in triplicate, meaning three separately prepared columns per sample. To purge any volatile contamination, we conditioned samples ahead of the respective experiment for one hour (at 0% RH and 10 cm^3^/min nitrogen flow, equated to the general measurement conditions). Furthermore, the double injection of methane to determine the dead volume at the beginning and the end was part of all experiments.

Prior to an SE assessment, we conducted the SSA determination by injecting octane concentrations (pressure range (*p/p*^0^) 0.05–0.35) leading to an adsorption isotherm. The SSA was hence calculated using the BET theory. Knowing the respective monolayer capacity of a sample allowed for SE determinations at specific fractional surface coverages. We used homologous alkanes from heptane to undecane as non-polar probes, as well as chloroform and toluene to determine acid–base properties of the sample. The injected concentrations led to specific surface coverages from 0.5% up to 20% of the monolayer capacity. The SEA analysis software (Surface Measurement Systems) enabled the calculation of the SE properties based on the raw data (retention of probes). All calculations were based on the DellaVolpe scale [19], the Dorris and Gray method (dispersive SE) and the polarisation method (specific SE) [20,21]. SE raw data were analysed regarding the peak centre of mass, whereas SSA calculations were based on peak maxima. SE data in this work are depicted as the average of three parallels with their respective standard deviation.

#### 2.2.6. Production of Interactive Blends

Interactive blends in this study were obtained by blending respective drug and carrier (and eventual extrinsic fines) in a high-shear mixer (Picomix^®^, Hosokawa Alpine) for 60 s twice at 500 rpm, with a sieving step in between (mesh size: 250 µm). All excipients underwent pre-process sieving at respective mesh sizes (drug: 180 µm; fines: 180 µm; carrier: 250 µm) to avoid large agglomerates. The production process of binary blends consists of weighing-in the drug (1% *w*/*w*) using the sandwich method, followed by the blending steps and a post-process sieving (mesh size: 250 µm). For ternary blends in turn, we added the fine excipient (7.5% *w*/*w*) in the first mixing step and the drug (1% *w*/*w*) within the second, complemented with post-process sieving (mesh size: 250 µm). The drug content was matched to earlier studies, and the respective batch size was 30 g [14,18]. High-performance liquid chromatography (HPLC) analysis of 10 randomly picked samples provided data on blend homogeneity. We considered a blend homogeneous at a drug recovery of 90%–110% and a relative standard deviation of less than 5%. Prior to further tests, we conditioned all homogeneous blends at 44% RH and room temperature for one week.

#### 2.2.7. Aerodynamic Assessment

Impaction analysis using the Next Generation pharmaceutical Impactor (NGI; Copley Scientific, Nottingham, UK) of conditioned blends provided information on aerodynamic particle size distributions and fine particle fractions (FPF) of the respective blends. FPF is defined as the percentage of the emitted dose with an aerodynamic diameter smaller than 5 µm. Delivered doses are defined as the mass of drug per shot, which was emitted from the device upon actuation. The blends were manually filled into reservoirs of Novolizer^®^ devices (MEDA Pharma, Bad Homburg, Germany). We set the flow rate through the NGI to 78.3 L/min to generate a pressure drop of 4 kPa over the inhaler and actuated the device 8 times per run. Each aerodynamic assessment consisted of three runs per blend, and all tests took place at controlled environmental conditions of 45% RH and 21 °C. Drug quantification was performed using HPLC. After quantification, we used CITDAS Software V3.1 (Copley Scientific) to calculate aerodynamic particle size distributions and hence resulting FPFs. NGI assessments were performed in triplicate; reported values are the respective averages in combination with the standard deviations.

#### 2.2.8. Quantification of Drug Content

The HPLC (Waters Corporation, Milford, CT, USA) method for fenoterol hydrobromide comprised using a reverse stationary phase (LiChrospher^®^ 100 RP-18, Merck, Darmstadt, Germany) and a solvent mixture consisting of 88% bi-distilled water adjusted to pH 3.0 (with phosphoric acid) and 12% acetonitrile as mobile phase. We conducted a method validation, which covered system suitability, specificity, precision, repeatability and linearity. The LOQ (limit of quantification) was 0.03 μg/mL (calculated according to the corresponding ICH guideline CPMP/ICH/381/95). Prior to every HPLC analysis, we freshly prepared an external standard calibration curve (0.2 μg/mL to 99.9 μg/mL, R^2^ > 0.99). All determined sample concentrations were within that calibrated range. We used chromatographic grade solvents for all HPLC procedures, which were purchased from Honeywell Riedel-de Häen (Chromasolv, Seelze, Germany).

The HPLC method for quantification of ipratropium bromide (including validation parameters) was previously reported [13,17].

## 3. Results and Discussion

### 3.1. Proof-of-Concept for Carrier Surface Energies Affecting Drug Detachment

#### 3.1.1. Creating a Portfolio of Different Carrier Surface Energies by Dry Particle Coating

**Figure 1:**
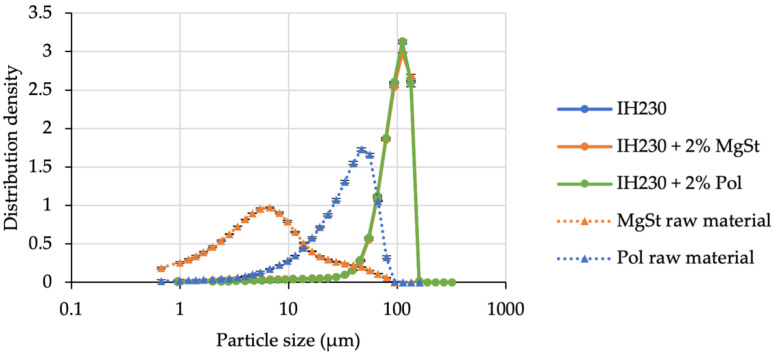
Particle size distribution of the carrier lactose with and without additives. *n* = 3, error bars denote standard deviation.

Prior to the blend preparation and the respective aerodynamic assessment, we characterised the compound carrier particles in terms of PSD and SE. Following the same protocol of previously published studies (utilising MgSt) [13], it was also possible to sufficiently coat the lactose carrier with poloxamer. Particle size distribution measurements served as verification for the firmly bound additive. Figure 1 shows the PSDs of InhaLac^®^ 230 before and after processing with magnesium stearate and the polar model additive (poloxamer 188) in their respective concentrations (2% *w*/*w*). The PSDs of the carriers being processed with an additive are similar to the unprocessed material indicating no substantial loose additive residuals in the powder, which would result in a shoulder in the PSD.

**Figure 2:**
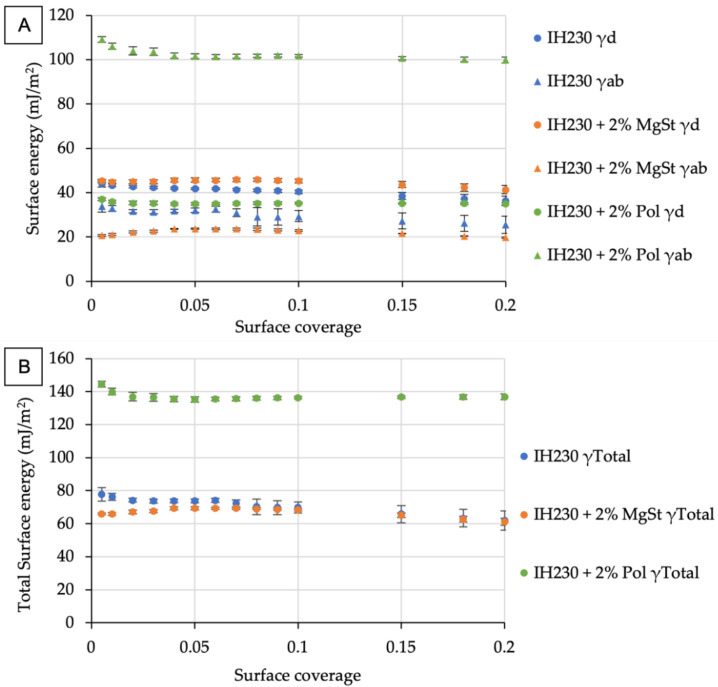
Surface energy distributions of lactose carriers with and without additives. (**A**) depicts the changes in the surface energy (γ) composition, i.e., the suffix “d” indicates the dispersive part and suffix “ab” the acid–base or polar part of the respective surface energy. (**B**) shows the resulting total surface energies for all samples. *n* = 3, error bars denote standard deviation.

The aim of this work was to demonstrate the technical feasibility of specifically altering the surface of a carrier and fines (see Section 3.2) and consequently the SE. Therefore, all samples were assessed using the Surface Energy Analyser to investigate process-induced SE modifications. In Figure 2, we plotted the respective dispersive, specific and total SE distributions of all three carrier batches. Figure 2A shows a small increase in dispersive SE for the MgSt-coated sample compared to the unprocessed and polar-coated lactose, which is due to the non-polar properties of MgSt. Moreover, Figure 2A displays an increase in specific SE of the polar-coated sample (green triangles), whereas the SE of the non-polar processed lactose indicates less polar surface properties (orange triangles). The distributions of the resulting total surface energies (Figure 2B) give a trend of the polar sample being the one with the highest SE levels, followed by the non-processed. The lowest total SE level was exhibited by the MgSt coated samples, which agreed with our previously published findings.

The absolute values of the specific surface energy, however, depend crucially on the probes used in the iGC method. Thus, specific SE and total SE provide comparative information, if the same methods were used, but are limited in deriving solid, value-based conclusions.

#### 3.1.2. Binary Blends for Inhalation Using the Engineered Carrier Particles

**Figure 3:**
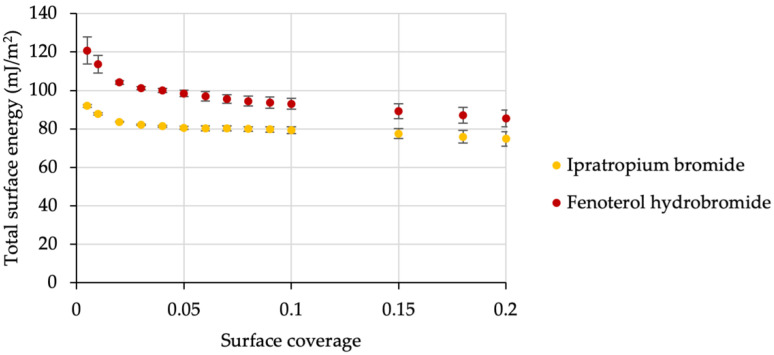
The distribution of total surface energies from the respective model APIs of this study. *n* = 3, error bars denote standard deviation.

The corresponding interactive blends of all carrier batches under investigation were then examined for their aerodynamic performances (FPF). We displayed results of the impaction analysis in Table 1. For ipratropium, being the model drug with a lower SE (Figure 3), the low-energy carrier performed significantly better than non-processed lactose (FPF increase of 145.7%, *p*-value < 0.05). The poloxamer-coated carrier resulted in a decreased FPF in comparison to the unmodified sample (decreased by 9.2%), although this is no statistically significant decrease (*p*-value > 0.05). De Boer et al. reported that the Novolizer device is comparably carrier-insensitive regarding the resulting FPF [22]. Thus, even if the carrier properties are detrimental for high drug delivery, the Novolizer device may cause relatively good FPFs, due to its effective dispersing principle [23]. We assume that there is a minimum (API-specific) FPF in this device, which we will not undercut by increasing thermodynamic adhesion. Although the decrease in FPF for the high-SE carrier is magnitudes lower than the increase for the low-SE carrier, this finding thus supports the hypothesis of higher SE interaction leading to stronger adhesion between the drug and carrier.

To verify this hypothesis, we also investigated the effect of using a drug with higher SE, namely fenoterol (Figure 3). According to Equation (1), the increase of the SE of any of both interacting surfaces (s1 and s2) will increase the overall SE interaction (SEI, WAdh) and hence the adhesion strength [24].

(1) $$W_{Adh}^{Total}=W_{Adh}^{dispersive}+W_{Adh}^{acid-base}=2\times \left[\sqrt{\gamma_{s1}^{d}\times \gamma_{s2}^{d}}+\sqrt{\gamma_{s1}^{-}\times \gamma_{s2}^{+}}+\sqrt{\gamma_{s1}^{+}\times \gamma_{s2}^{-}}\right],$$

Figure 3 displays the surface energy distribution of fenoterol hydrobromide in relation to ipratropium bromide. The overall higher surface energy level should cause stronger interaction with the carrier particle Equation (1).

All resulting FPFs of interactive blends based on the above-mentioned carriers formulated with fenoterol as the API were lower compared to the ones using a lower-energy drug (ipratropium bromide; Table 1). Aiming for a decreased FPF has no practical use but serves as a proof of the general influence of SE alterations.

We conclude, if the SEI is increased in a binary system, the FPF will decrease due to increased adhesion between the drug and carrier. This can be achieved by either exchanging one of the substances or altering their surface.

### 3.2. Particle Engineering of Extrinsic Fines Enables Surface Energy Matching with the Drug

#### 3.2.1. Evaluation of Particle Engineering Methods

According to our preceding work, SE differences (induced by manufacturing route) of fine excipients will cause differences in the resulting FPFs of ternary blends for inhalation. In our extensive study of a range of fine lactose qualities, we found that a higher SE of fines led to higher FPFs of the respective adhesive blends. We explained the results with a higher agglomeration tendency of the high-energy fines with the drug and more efficient saturation of the active sites. Therefore, particle engineering on the fine additives level (here InhaLac 400) should probably enable the extension of that beneficial effect.

**Figure 4:**
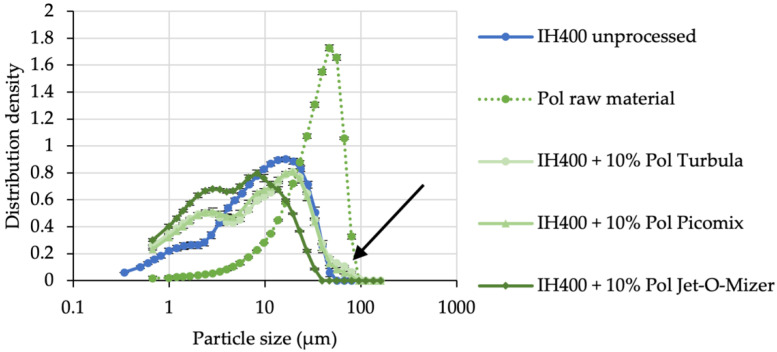
Resulting PSDs from the evaluation of particle engineering methods, which are unsuitable for extrinsic fines (exemplary shown for poloxamer processes). Turbula blender as low-shear blending is shown in light green circles, Picomix as exemplary for high-shear mixing is depicted in medium green triangles and the Jet-O-Mizer as co-milling approach is coloured in dark green squares. The black arrow points at the shoulder in PSD as a result of loose, residual additive. *n* = 3, error bars denote standard deviation.

Previously reported engineering methods (i.e., high-shear mixing, green triangles in Figure 4) were not suitable to coat high amounts of additive onto the surface of fine lactose. The PSDs of InhaLac 400 processed with 10% additive (*w*/*w*) indicated an insufficient coating by a shoulder (black arrow) matching the d_50_ of pure poloxamer 188 at approximately 30 µm (Figure 4).

**Figure 5:**
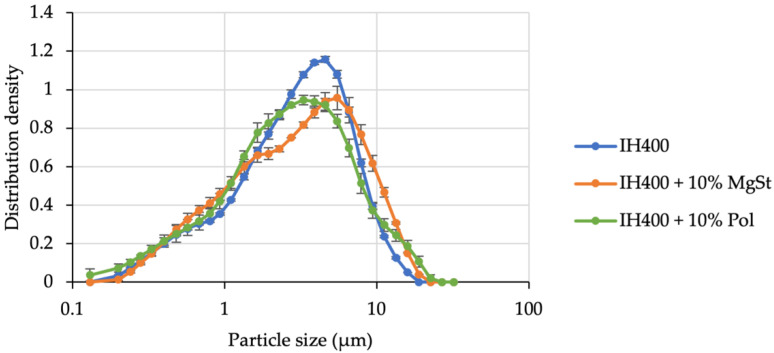
Particle size distribution after the optimisation of grinding pressures in the air jet mill. *n* = 3, error bars denote standard deviation.

Thus, we evaluated another particle engineering method, which is more suitable to produce compound fines. Co-milling is capable of applying high energy (particle–particle and particle–wall collisions) and hence merging or coating [25,26] of the substances in the grinding chamber. After optimising the grinding conditions, the process resulted in similar particle sizes (Figure 5).

**Figure 6:**
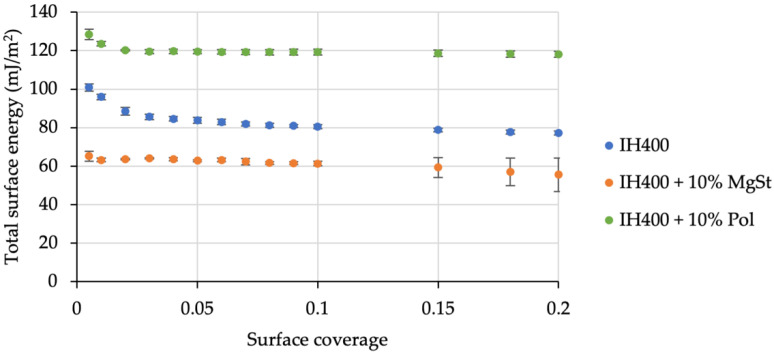
Distribution of total surface energies of fines engineered by co-milling. *n* = 3, error bars denote standard deviation.

To test our hypothesis, we measured the respective SE distributions of the novel compound fines. As is clearly visible in Figure 6, processing with either high- or low-energy additives led to crucial changes in the fines’ SE levels. Showing the same trends as for processed carriers, merging with MgSt resulted in decreased total surface energies, whereas co-milling with poloxamer led to significantly increased SE.

#### 3.2.2. Ternary Blends for Inhalation Using the Engineered Fines

Within a ternary blend, a high-energy fine particle should exhibit higher adhesion forces and hence result in increased saturation of active sites and increased agglomeration with the API. These agglomerates are known to increase drug detachment from the carrier and to result in higher fine particle fractions, if readily dispersible [27].

Therefore, we determined the FPF of the respective ternary blends, containing the novel compound fines (Table 2). Contrary to our expectations, the blends with fines processed without an additive resulted in the highest FPF. Both compound fines performed worse, with the high-energy fines being statistically significantly inferior to the additive-free fines (*p*-value < 0.05).

All ternary blends were additionally prepared using fenoterol hydrobromide as the API with higher SE levels (Figure 3). The FPFs decreased again compared to ipratropium bromide blends. This finding further substantiates the hypothesis that a higher SE of drugs results in higher adhesion and hence less drug detachment and the formation of API–fines agglomerates, which are hard to disperse. The only exception was the ternary blend containing MgSt, which showed higher FPF for fenoterol than for ipratropium. All blends prepared with fenoterol differed statistically significantly from each other (*p*-value < 0.05). Since the combination of the low-energy fines with the high-energy API resulted in the best performance, it seems reasonable that there is an optimal SEI for a given drug–carrier combination and device.

This is further substantiated by evaluating statistically differences of FPFs between the respective drugs per fine quality. The high-energy drug (fenoterol) performed significantly better (*p*-value < 0.05) in combination with low-energy fines (MgSt). Ipratropium bromide as a low-energy drug in turn reached significantly higher FPFs (*p*-value < 0.05) combined with high-energy fines (Pol-processed). Both drugs led to FPFs without statistically significant differences (*p*-value > 0.05) if formulated with non-modified (medium-energy) fine excipients.

The drug is usually a fixed parameter in formulation development, and modifications should be evaluated extremely critically. As the specific alteration of drug particles by particle engineering could result in changes in the respective clinical effect, scientists typically focus on excipient modifications. The obtained data indicated that matching the drugs’ SE with the SE of fines could be beneficial. In doing so, a high SE drug may be compensated with low SE fines, for instance. Furthermore, the co-milling approach we introduced in this study enables the tailoring of fines in dependence of the drugs’ SE.

**Figure 7:**
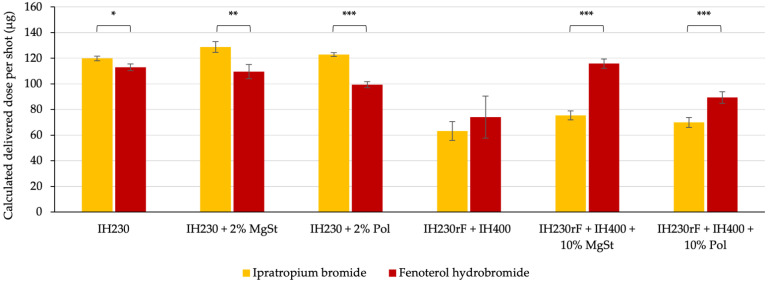
Calculated delivered doses of all assessed formulations in µg. *n* = 3, error bars denote standard deviation. Significance depicted as: * *p*-value < 0.05; ** *p*-value < 0.01; *** *p*-value < 0.005.

Additionally, we assessed the changes in delivered doses (DD) when switching from ipratropium (lower SE) to fenoterol (higher SE) in Figure 7. The DD decreased in the binary blends due to increased adhesion forces between the carrier and drug. The trend in DD matches the trend in FPFs. In ternary blends, in contrast, the DD increased in fenoterol blends, even though the FPF decreased. This is due to a higher emission of the drug, probably based on more drug–fine agglomerates, which detach more easily from the carrier but are hardly dispersed into individual particles < 5 µm.

## 4. Conclusions

Based on the experimental data, we conclude that systematically modified surface energies in binary blends work according to fundamental adhesion (thermodynamic adhesion) theories. This means that both an increased adhesion of the drug by an SE increase of the carrier and a decreased adhesion of the drug to the carrier by reduction of the carrier SE are feasible. We showed that a decreased carrier SE and thus decreased adhesion after processing with MgSt is not just an artefact but a causal relationship. This highlights SE assessments to keep track of changed adhesion properties after particle engineering. Substantiating the influence of thermodynamic adhesion, changing the drug to one with higher SEs also resulted in decreased drug detachment.

Engineering of high- and low-energy extrinsic fines for DPI formulations was also feasible, but the results of aerodynamic assessment were not fully matching earlier stated hypotheses. Usually, practical adhesion forces are larger than just thermodynamic adhesion forces (surface energy interactions) [28]. Thus, trends in SE will just transfer to trends in adhesion if a major part of the particle properties does not change. We assume that by fundamentally altering the particle properties by jet mill processing, the properties influencing practical adhesion (not SE related, such as plastic or viscoelastic deformation upon the break of the adhesive joint) changed crucially. Therefore, earlier observed correlations of fines SEs and respective FPFs are not extendable due to practical adhesion.

Nonetheless, we showed that high-energy fines performed better with a low-energy drug and vice versa. This substantiated the hypothesis of SE matching as a strategy to enhance the drug delivery of ternary blends.

In general, it is not possible to correlate DPI performance with just single solid-state parameters directly. Our study emphasises the influence of the SE on adhesion, which translates into an influence in drug detachment and delivery. In a multifactorial system such as DPI formulations, we must take several influencing parameters into account to enable a more purposive formulation development and optimisation.

Particle engineering in DPI formulations holds great potential to optimise overall performance. Even though modification techniques on the carrier level are well established, the engineering of extrinsic fines is a novel approach. Additional experiments and method optimisations are needed to fully develop this approach and exploit its potential towards enhanced respiratory drug delivery. The findings of this study underline the importance of surface energy considerations within DPI formulation development.

## Figures and Tables

**Table 1:** Calculated FPFs at a 5 µm cut-off, derived from NGI assessments. *n* = 3, standard deviation in parentheses.

Carrier	FPF Ipratropium (%)		FPF Fenoterol (%)	
IH230	23.2	(1.9)	11.9	(2.1)
IH230 + 2% MgSt	57.0	(0.1)	56.1	(0.6)
IH230 + 2% Pol	21.1	(1.6)	11.1	(0.7)

**Table 2:** FPF at a 5 µm cut-off for ternary blends containing engineered fines. “IH400” herewith means that the fines are based on the processed IH400 quality, if stated, with the respective additive. *n* = 3, standard deviation in parentheses.

Carrier Blend	FPF Ipratropium (%)		FPF Fenoterol (%)	
IH230rF + IH400	50.5	(2.4)	46.9	(1.3)
IH230rF + IH400 + 10% MgSt	47.9	(2.5)	62.9	(0.8)
IH230rF + IH400 + 10% Pol	45.0	(1.8)	38.9	(2.5)
